# Periprosthetic bacterial and fugal infection after total knee arthroplasty with one-stage debridement: a case report

**DOI:** 10.1186/s13256-024-04492-5

**Published:** 2024-04-13

**Authors:** Yujiang Liu, Junxin Lin

**Affiliations:** 1 Department of Spinal Surgery, Qingdao Traditional Chinese Medicine Hospital (Qingdao Haici Hospital), Qingdao, 266000 Shandong China; 2 Department of Joint Surgery, Qilu Hospital of Shandong University (Qingdao), Qingdao, 266000 Shandong China; 3 Key Laboratory of Qingdao in Medicine and Engineering, Qilu Hospital of Shandong University (Qingdao), Qingdao, 266000 Shandong China

**Keywords:** Fungal infection, Knee arthroplasty, Complication, Debridement

## Abstract

**Background:**

Periprosthetic infection is a serious complication after arthroplasty and is characterized by a long duration, recurrence, and a low cure rate. Although fungal infections are infrequent, they are often catastrophic, with an insidious onset, a long duration, atypical clinical symptoms, and imaging features in the early stage. They are easily misdiagnosed, or the diagnosis is missed, resulting in wrong treatment approaches.

**Case presentation:**

This paper reports a case involving a 62-year-old female patient of Korean ethnicity with a periprosthetic infection after knee arthroplasty who underwent joint debridement. A preoperative metagenomic next-generation sequencing of joint aspirate revealed *Staphylococcus epidermidis*. However, postsurgical tissue cultures confirmed the fungal infection. The patient received oral voriconazole and intra-articular injection of voriconazole for antifungal treatment. Since bacterial infection could not be ruled out, we also prescribed levofloxacin. No infection recurrence was observed after more than 22 months of follow-up. In the treatment of this patient, successful short-term follow-up was achieved, but long-term efficacy still cannot be determined.

**Conclusions:**

In addition to the case study, we provide an analysis of the diagnosis and treatment of fungal infection after arthroplasty, especially the efficacy of debridement, antibiotics, and implant retention for a short-term outcome.

## Background

Periprosthetic infection is a serious complication after arthroplasty and is characterized by a long duration, recurrence, and a low cure rate. Although fungal infections are infrequent, they are often catastrophic, with an insidious onset, a long duration, atypical clinical symptoms, and imaging features in the early stage. They are easily misdiagnosed, or the diagnosis is missed, resulting in wrong treatment approaches. Herein, we report the treatment procedure, that is, debridement, antibiotics, and implant retention (DAIR), for one patient with a fungal infection after knee arthroplasty who was admitted to and treated at the department of joint surgery at Qilu Hospital of Shandong University (Qingdao) in March 2021. The shortcomings and current understanding of the diagnosis and treatment of the disease are discussed in combination with a literature review to provide references for the diagnosis and treatment of this disease.

## Case presentation

**Fig. 1:**
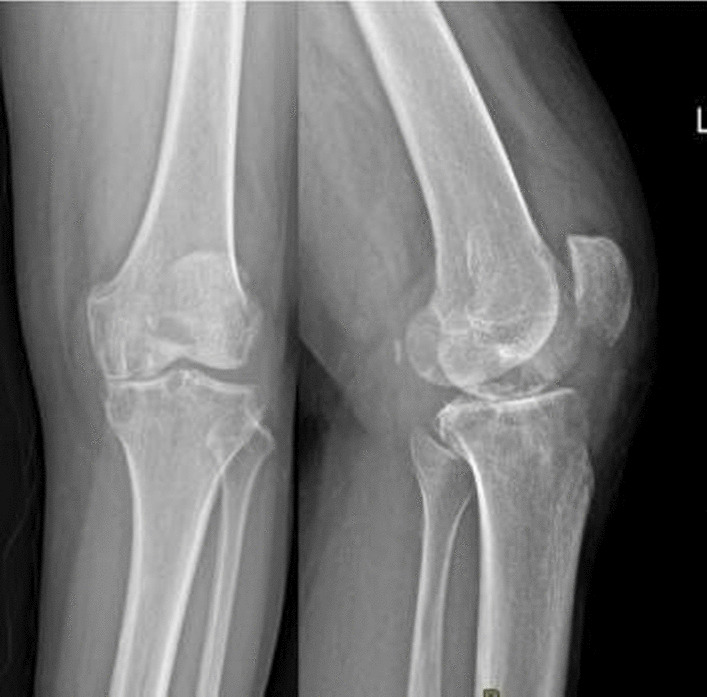
X-ray showing obvious cystic changes in the proximal tibia and distal femur of the left knee joint

**Fig. 2:**
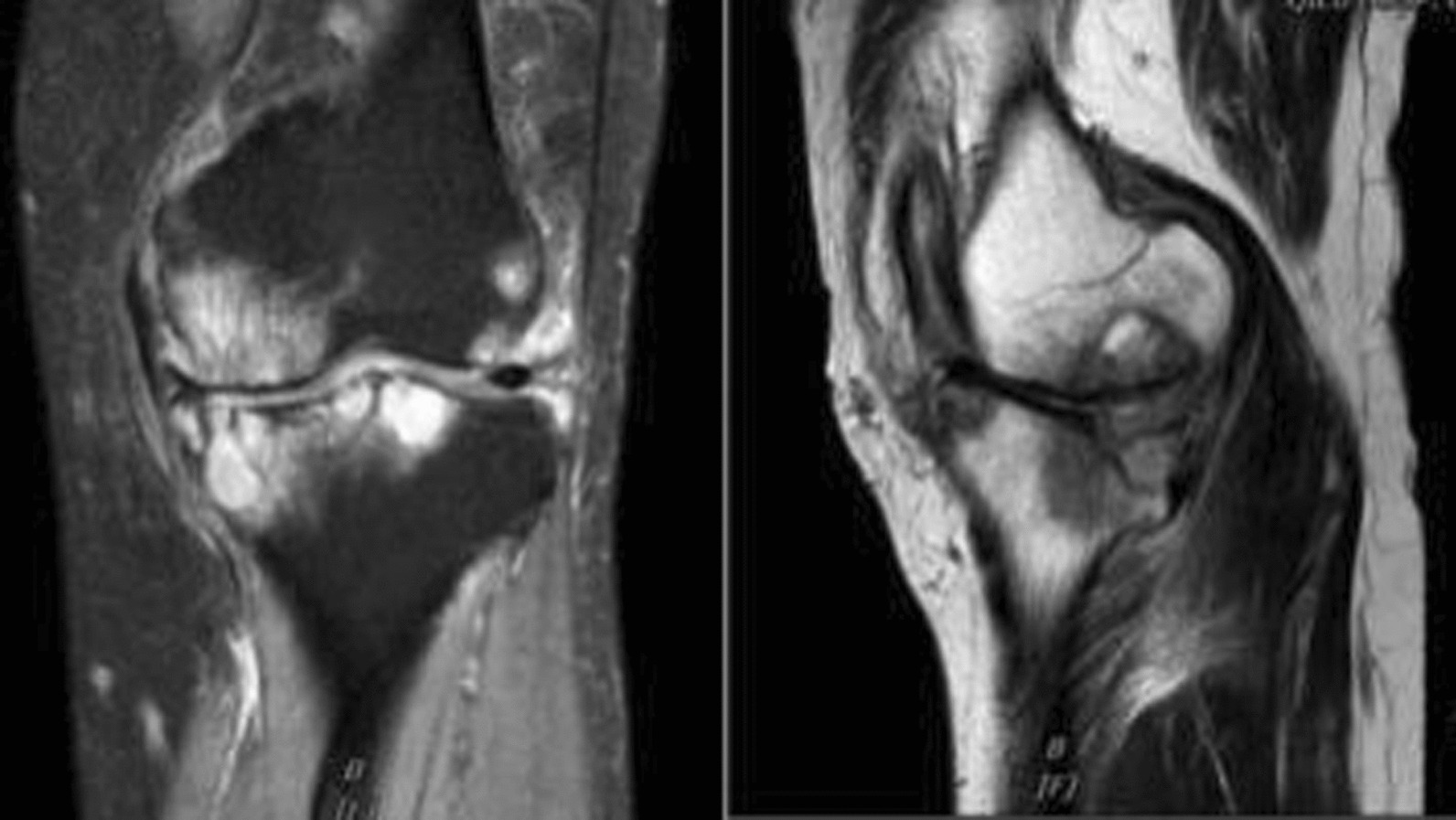
Magnetic resonance imaging (MRI) of the left knee showing multiple cystic changes in the distal femur and proximal tibia, bone marrow edema, and no inflammatory changes in the soft tissues surrounding the knee joint

**Fig. 3:**
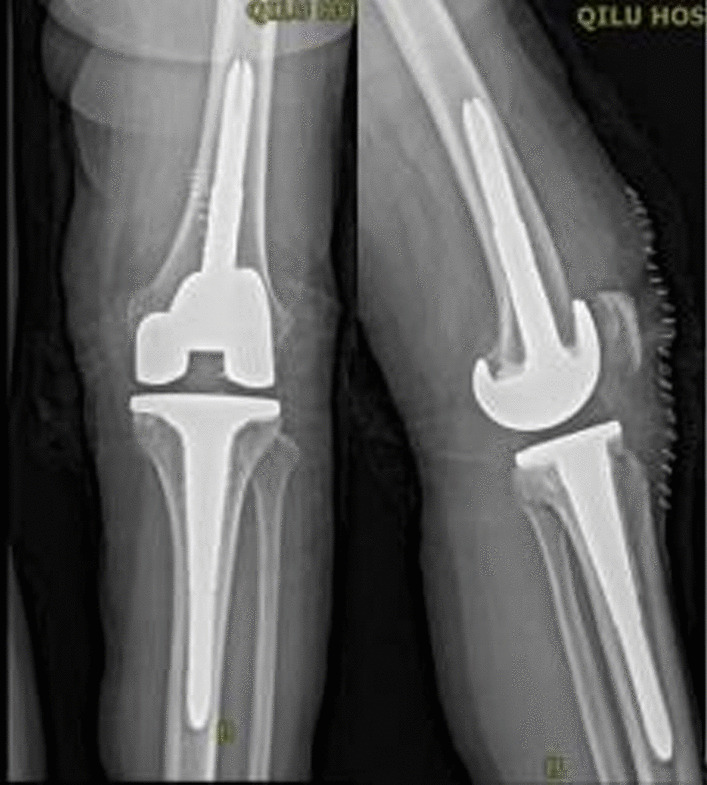
Cystic changes were eliminated after left knee arthroplasty

**Fig. 4:**
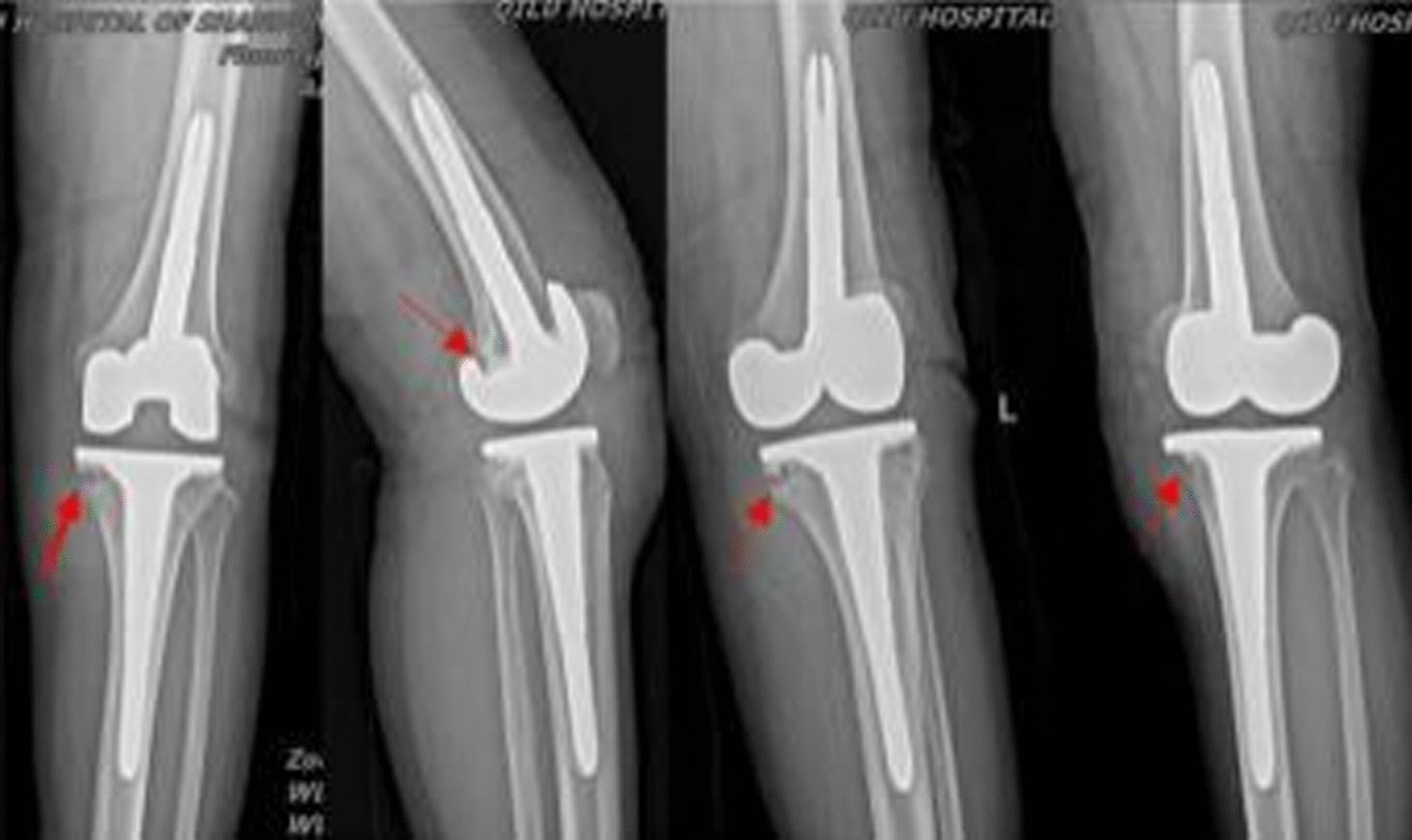
The anteroposterior, lateral, and double oblique radiographs of the left knee joint reveal translucent zones (indicated by red arrows) at the femoral posterior condyle and under the tibial prosthesis

A 62-year-old woman of Korean ethnicity suffering from left knee arthralgia was hospitalized and diagnosed with left knee osteoarthritis complicated with grade 2 hypertension, type 2 diabetes mellitus, and tinea pedis. In October 2020, after her blood glucose was stabilized, the patient underwent bicompartmental arthroplasty of the left knee performed by an experienced chief physician in our hospital, during which a Depuy MBT joint prosthesis was used. Obvious cystic changes around the knee joint were observed in the preoperative imaging findings (Figs. 1, 2), and severe osteoporosis and contained bone defects in the medial tibial plateau were discovered after osteotomy during surgery; therefore, femoral and tibial stem extension implants were placed during surgery (Fig. 3). The drainage tube was removed within 24 hours, and prophylactic antibiotics were applied within 24 hours of the perioperative period. At 10 hours after surgery, enoxaparin [40 mg once per day (qd)] was administered but was later changed to rivaroxaban (10 mg qd) to prevent lower-extremity venous thrombosis after discharge. The patient recovered a good joint range of motion after surgery, and she was discharged 5 days after the operation. The incision healed well, and the suture of the stage 1 incision was removed 14 days after surgery. Her erythrocyte sedimentation rate (ESR) and C-reactive protein (CRP) concentration were 11.00 mm/hour and 2.1 mg/L, respectively, before the operation and 10.00 mm/hour and 59.21 mg/L, respectively, 3 days after the operation. The patient complained of pain at the pes anserinus of the anteromedial knee during outpatient reexaminations at 2 weeks, 1 month, 2 months, and 3 months after surgery, and she received symptomatic treatment with anti-inflammatory and analgesic drugs. The patient was reexamined approximately 4 months after surgery owing to aggravated pain and was found to have mild local redness and swelling of the medial tibial tubercle of the left knee and a slightly elevated skin temperature. The patient was reexamined, and her ESR and CRP concentrations were 21.00 mm/hour and 14.90 mg/L, respectively. Oral levofloxacin (500 mg qd) was prescribed for suspected bacterial infection, but a mass (approximately 2 × 2 cm^2^) appeared at the medial tibial tubercle 1 month later, with no obvious local fluctuation and knee range of motion of approximately 15–70°. The patient was admitted to the hospital, and infection after knee arthroplasty was considered. After admission, her ESR and CRP concentrations were 23.00 mm/hour and 10.04 mg/L, respectively, and an ultrasound-guided knee puncture was performed, during which a small amount of dark red fluid was obtained for bacterial and fungal culture. The results of metagenomic next-generation sequencing (mNGS) indicated *Staphylococcus epidermidis*. X-ray of the left knee showed abnormal translucent zones under the medial tibial plateau and at the femoral posterior condyle (Fig. 4).

**Fig. 5:**
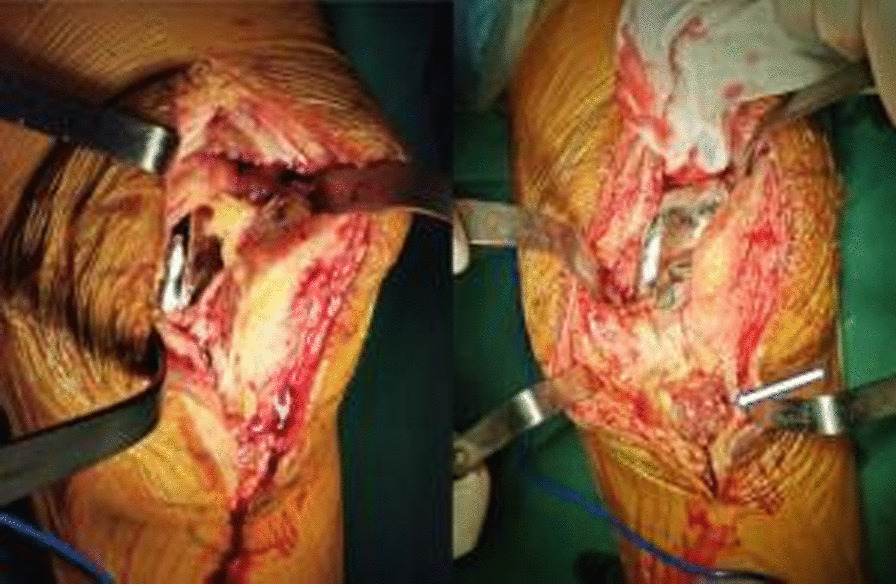
Papillary granulomatous hyperplasia in the joint was observed during surgery, without purulent joint fluid but with a local unruptured granulomatous mass (indicated by the white arrow) at the medial tibial tubercle

**Fig. 6:**
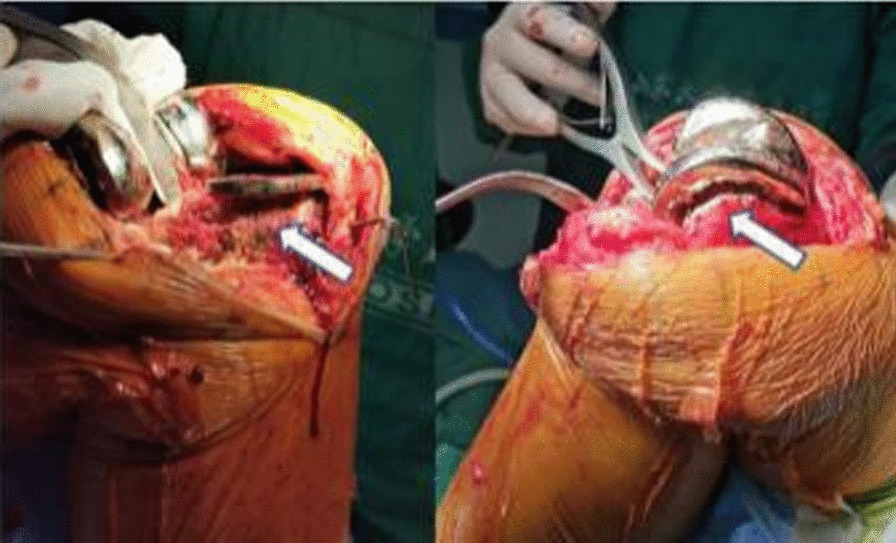
Surgery revealed local cystic changes under the tibial plateau and at the femoral posterior condyle, local bone defects (indicated by white arrows), and no prosthesis loosening

**Fig. 7:**
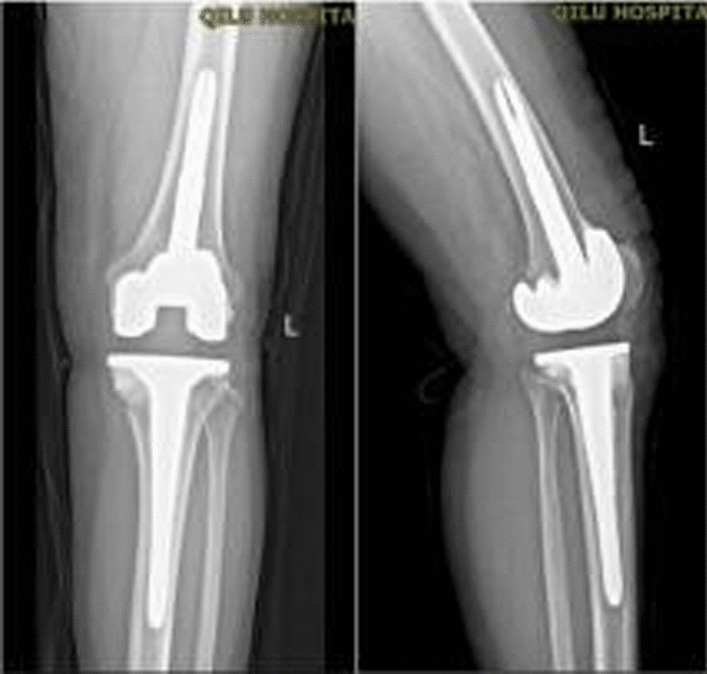
Imaging results at 22 months after surgery

In March 2021, the patient was initially diagnosed with acute infection after knee arthroplasty because the symptoms of knee infection emerged in less than 3 months, and DAIR was considered. Intraoperative exploration revealed an unruptured local granulomatous mass at the medial tibial tubercle (Fig. 5), which was excised. There was no apparent joint fluid in the knee, no remarkable loosening of the prosthesis, no significant wear of the liner, and massive papillary synovial hyperplasia. Hence, joint dislocation was performed first, and the liner was removed. Then synovectomy and adhesiolysis were conducted. During the debridement, local cystic changes under the tibial plateau and the femoral posterior condyle were observed; there were local bone defects, but the prosthesis was not loosened (Fig. 6). The bone defects were filled with bone cement (40 g of bone cement with 1 g of vancomycin) to eliminate spaces, and the knee range of motion was 0–110°. After surgery, intravenous drip infusion of vancomycin (1 g every 12 hours) and rifampicin (0.45 g orally qd) was continued. Intra-articular injection of vancomycin (1 g qd) was administered in a dressing room 48 hours after surgery. Standard procedures for knee puncture were strictly followed to minimize the risk of contamination or reinfection of the knee. At 4 days after surgery, the culture results of specimens obtained before and during surgery were positive for *Candida parapsilosis*. Therefore, voriconazole [200 mg twice per day (bid)] and levofloxacin (500 mg qd) were administered orally. The intra-articular injection of vancomycin was stopped and replaced with an intra-articular injection of 200 mg voriconazole dissolved in 10 mL saline once daily for 6 consecutive weeks. Additionally, joint fluid conditions were observed, gradually turning clear from turbid. A routine test of the joint fluid was performed weekly until the white blood cell count was less than 3000 and the neutrophil ratio was less than 75%. After discharge, the patient continued with oral voriconazole (200 mg bid) and levofloxacin (500 mg qd) until 6 months after surgery. After 22 months of postoperative follow-up, no remarkable signs of loosening were indicated on the plain radiograph of the knee joint (Fig. 7), and the patient’s knee range of motion recovered to 0–110°, without knee arthralgia or abnormalities in liver and kidney function.

## Discussion and conclusions

As the most serious complication following arthroplasty, periprosthetic joint infection (PJI) is characterized by strong destructiveness, complex treatment, and a low cure rate. *Staphylococcus* is the most common PJI pathogen, and *Staphylococcus*-induced PJI accounts for approximately 50–60% of PJIs after arthroplasty [1]. Fungal PJIs are less common, accounting for approximately 1% of PJIs, with *Candida* (*Candida albicans* or *Candida parapsilosis*) being the major pathogenic fungus [2]. Fungal PJIs may lead to devastating damage to the joint if not treated in time, making the diagnosis and treatment of this disease challenging [1].

Unlike bacterial PJIs, fungal PJIs have atypical clinical symptoms, without typical redness, swelling, fever, and pain. X-ray evidence indicates that the disease has a relatively slow and insidious onset, causing some difficulties in clinical diagnosis. The main manifestations of fungal infection after arthroplasty on X-ray mainly include local soft tissue swelling, apparent osteoporosis, local bone destruction, and progressive translucent zones. Fungal PJIs often occur in patients with multiple chronic medical diseases or immunodeficiency. Khalid *et al*. [3] reported 31 cases of patients with fungal PJI diagnosed between 1999 and 2006. Among them, 27 patients had one or more chronic medical comorbidities (including chronic liver disease, tumors, rheumatoid arthritis, and diabetes mellitus), 6 patients were treated with glucocorticoids, and 7 patients had a history of long-term antibiotic use before fungal PJI. In a study by Zou [4], six (50.0%) patients had three or more medical diseases, and seven patients had received two or more surgical treatments. These factors, which affect the immune function of patients, have significant impacts on the treatment of fungal PJIs, further increasing the difficulty of treatment. The patient in this paper was at high risk of infection because she had type 2 diabetes mellitus and advanced age. Her blood glucose was not well-controlled at admission. After her blood glucose was stabilized, the patient immediately underwent surgery. The decrease in autoimmunity caused by various diseases may be the systemic basis for fungal infection after arthroplasty.

Currently, the basic treatment methods for PJIs include simple antibiotic therapy, DAIR, two-stage joint revision, one-stage joint revision, postoperative arthroplasty, and amputation [5, 6]. Simple antibiotic therapy can merely inhibit bacterial development and is unable to completely eradicate deep infection around the prosthesis. Therefore, it is not appropriate to use antibiotics alone to treat PJIs after arthroplasty [7]. It is difficult to treat fungal PJIs in the clinic owing to characteristics, such as the rapid formation of complex biofilms on the prosthesis surface and the rapid development of drug resistance, in addition to the low immune status of the host. The overall mortality rate of *Candida* PJIs is as high as 25% [1]. Gross *et al*. [8] reported that the success rate of prosthesis retention in treating fungal PJIs is 15 %, and Escolà-Vergé *et al*. reported that the success rate of DAIR for fungal infections is only 27% [9]. Currently, according to the recommendations of the Infectious Diseases Society of America (IDSA) and the Institute for Complementary Medicine (ICM), fungal PJIs should be treated with two-stage joint revision combined with systemic and local antifungal drugs as soon as possible, antifungal treatment should be carried out for at least 6 weeks, and the course of treatment should be extended as much as possible after surgery. With improvements in diagnostic techniques, the success of one-stage revision has been widely reported. For example, Ji *et al*. [10] reported 7 out of 11 cases of fungal PJI successfully treated with one-stage revision. A study by Zou [4] also reported good results of one-stage revision for fungal PJIs. Some scholars have even proposed three-stage revision arthroplasty, with local and systemic antifungal treatments for optimizing the treatment of fungal PJIs [11]. Although DAIR is unfavorable for biofilm recurrence, it is a less disruptive surgical method to save the implant. A multicenter retrospective study showed that previously failed DAIR did not compromise the success rate of a subsequent staged revision [12]. The results of DAIR are highly dependent on isolating microorganisms and antibiotic-sensitive cultures. The intra-articular injection of voriconazole could have increased the drug concentration in the joint and reduced systemic toxicity, but the risk of bacterial contamination should be noted.

In this case, local redness and swelling of the knee joint lasted for less than 2 months. Moreover, the prosthesis exhibited good stability despite evident bone defects in the periphery. We considered the patient to have an acute postoperative infection, which originated from hematogenous infection. The DAIR procedure could be controversial owing to the course of the disease and the pathogen. The preoperative diagnosis of PJI was inconsistent with the results of the postoperative tissue culture. Postoperative tissue and joint fluid cultures confirmed *Candida parapsilosis* infection, and *Staphylococcus epidermidis* was detected in only one preoperative genetic test, for which the possibility of specimen contamination before surgery was considered. Hence, sensitive antifungal drugs were adopted on the basis of the fungal culture results. Given the high bioavailability, good tissue penetration and bone permeability, and relatively stable minimum inhibitory concentration, voriconazole was orally administered in strict accordance with its half-life, and 200 mg of voriconazole was regularly intra-articularly injected for 6 weeks, followed by continuous oral administration of voriconazole until 6 months after surgery. At 3 weeks after surgery, the results of multiple bacterial cultures of joint fluid were negative. Although satisfactory short-term efficacy was obtained, the case cannot be used as a basis for the clinical promotion of DAIR and may serve as a reference for patients diagnosed with fungal PJI after DAIR without definitive preoperative pathogen diagnosis. In addition, the patient in this case study was treated with levofloxacin prior to the joint puncture, which may affect the culture results and should be avoided.

In summary, destroying biofilms is the focus of PJI treatments, as fungi are capable of rapidly forming refractory biofilms. Therefore, the natural biological barriers of fungi were removed via thorough debridement, thus laying a good foundation for subsequent drug therapies. Sensitive antibiotic treatment was promptly adjusted after identifying the pathogenic fungus, and local intra-articular injection combined with systemic antifungal drugs was administered. In addition, systemic treatment for the patient was adjusted, and her liver and kidney function was examined regularly. In the treatment of this patient, successful short-term follow-up was achieved, but long-term efficacy still cannot be determined. Long-term follow-up and treatment tracking will be conducted.

## Data Availability

Not applicable.
